# Weakly supervised deep multi-instance learning for classification of endometrial lesions on hematoxylin and eosin-stained whole-slide images

**DOI:** 10.1371/journal.pone.0340186

**Published:** 2026-01-02

**Authors:** Zhixian Zhou, Jiaqi Guo, Xianglong Du, Hansheng Li, Nian Xie, Chengniang Liu, Hao He, Lin Yang, Lei Cui, Chun Fu

**Affiliations:** 1 Department of Obstetrics and Gynecology, The Second Xiangya Hospital of Central South University, Changsha, China; 2 Clinical Research Center for Gynecological Disease in Hunan Province, Changsha, China; 3 School of Information Science and Technology, Northwest University, Xi’an, China; 4 School of Engineering, Westlake University, Hangzhou, China; Korea Institute of Radiological and Medical Sciences, KOREA, REPUBLIC OF

## Abstract

Endometrial cancer (EC) is the most common gynecological malignancy, yet reliable screening and diagnostic approaches remain limited. We developed a weakly supervised deep multi-instance learning model (DSMIL) to classify hematoxylin and eosin-stained whole-slide images (WSIs) of endometrial tissue. A total of 885 WSIs from 442 patients, including EC, atypical endometrial hyperplasia (AEH), endometrial hyperplasia without atypia (EH), and normal endometrium (NE), were analyzed. DSMIL achieved an average AUROC of 0.9776 for four-class classification, with inter-class AUROCs of 0.9876 for EC, 0.9600 for AEH, 0.9771 for EH, and 0.9855 for NE, and outperformed other algorithms such as TransMSL, CLAM, and ABMIL (average accuracy = 0.8914). Attention heatmaps highlighted regions associated with pathological features, while nnU-Net v2 combined with HoverNet enabled identification of atypical glandular epithelial cells, which showed increased density, size, and perimeter but reduced axis ratios compared with normal cells. These results suggest that DSMIL provides a reliable computational pathology approach for the classification of endometrial lesions and the characterization of atypical cells.

## 1. Introduction

Endometrial cancer (EC) is the most common gynecological cancer among women worldwide, and its incidence and mortality are on the rise [1,2]. There are currently no mature and effective screening methods for EC [3–6]. It is crucial to identify endometrial lesions early and accurately so that accurate treatment options can be selected and fertility can be preserved, improving patients’ quality of life and reducing EC risk. As of today, the gold standard method of diagnosing endometrial lesions remains histopathological biopsy classification [7,8]. The shortage of pathologists and differences in human subjectivity may lead to a certain misdiagnosis rate in the diagnosis of endometrial lesions. Therefore, the introduction of artificial intelligence (AI) assisted diagnosis technology in our study will effectively reduce subjective errors and improve diagnostic efficiency and accuracy, thus facilitating early clinical precision diagnosis and treatment of endometrial diseases.

EC is commonly caused by endometrial hyperplasia as a precursor. It is classified by the World Health Organization (WHO) into two types: atypical endometrial hyperplasia (EH) and atypical endometrial hyperplasia (AEH) [9]. Women with EH are more likely to seek progesterone therapy to preserve their fertility [10,11]. AEH is treated radically by hysterectomy, as up to 40% of EC patients also have AEH [12–14]. Consequently, it is very critical to diagnose endometrial lesions early and accurately to determine the proper treatment course. Affected by the variation of the menstrual cycle and the differing degrees of disease progression, the pathological structure of endometrial tissue has obvious individual variations and structural complexity, which makes diagnosis with artificial vision in pathology significantly more challenging. We developed AI algorithms for analyzing endometrial pathological characteristics, such as glandular/interstitial distributions and changes in glandular epithelial cytology [15,16]. The use of these procedures allows patients with endometrial disease to benefit from more accurate clinical diagnosis and treatment, and to enjoy a more positive health care experience overall.

Many supervised deep learning models have been applied to endometrial cancer in recent years, contributing to the advancement of AI-assisted diagnosis [17–20]. These methods require high-quality manual annotations to supervise model training, which is very time-consuming and resource-intensive [21]. Weakly supervised learning can be used to train unlabeled hematoxylin and eosin (HE)-stained whole-slide images (WSIs) of endometrial cancer, which minimizes the reliance on large labeled datasets, such as the variable-aware multi-instance learning (MIL) method [22]. In a recent study, Wang et al. applied the classical MIL method to assess the risk associated with specific molecular subtypes of EC, which shows high accuracy and a short inference time, thus demonstrating clinical relevance [23]. However, the majority of current studies focus primarily on the molecular subtypes of endometrial cancer [17,21–24], ignoring the prodromal stages of EC. We need to focus more on the accurate and efficient diagnosis of endometrial prodromal lesions to provide effective treatment for patients with endometrial lesions, reduce the risk of EC occurrence, and, in particular, protect the fertility of patients who require fertility treatment.

Our study uses a weakly supervised MIL framework to extract endometrial histopathologic image features for rapidly and accurately distinguishing EC, AEH, EH, and normal endometrium (NE). Using the attention mechanism, the MIL framework can quickly identify the concentrated tissue area that contains pathological features. This will enable pathologists to reduce section reading times and test the algorithm’s diagnostic accuracy. Further validation of the pathology-related interpretability of the MIL working model was performed by analyzing glandular structure and glandular epithelial cell atypia in key regions. The model has been demonstrated to be accurate in identifying atypical cells in AEH and EC, making it useful for shunting patients requiring surgical treatment in clinical diagnosis and treatment. In this study, a digital pathological work network was introduced to assist in diagnosing and treating endometrial diseases more quickly, efficiently, and accurately.

## 2. Materials and methods

### 2.1. WSIs datasets

This retrospective study was approved by the Medical Ethics Committee of the Second Xiangya Hospital of Central South University (approval number: LYF2022231). The consent was waived by the ethics committee. From 01/02/2023–31/12/2023, a total of 442 formalin-fixed and paraffin-embedded endometrial samples were collected from diagnostic curettage or hysterectomy at the Second Xiangya Hospital of Central South University. The samples and associated clinical data were accessed for research purposes on 02/01/2024. Authors had access to identifiable patient information during the initial data collection; however, all data were fully anonymized before analysis, and no identifiable information was available thereafter.

After the data collection is completed, the relevant private information of individual participants cannot be identified. WSIs of hematoxylin and eosin (HE) stained endometrial pathological sections were produced using a tissue scanner (SQSL-510) at 400X magnification for subsequent analysis. A total of 885 endometrial pathology slides were used, divided into four categories: 442 slides of EC (131 patients), 198 slides of AEH (131 patients), 146 slides of EH (100 patients), and 99 slides of NE (99 patients). The slides were divided into three independent sets: training, validation, and testing, with 535 slides in the training set, 175 slides in the validation set, and 175 slides in the test set. Due to the high resolution of the pathology images, it was not feasible to directly extract features from them. This paper first used the NOBUYUKI OTSU Method (OTSU) [25] algorithm to separate the effective tissue regions of the pathology images, then cut the tissue regions into 256-size blocks under 20x magnification.

### 2.2. Methods

**Fig 1:**
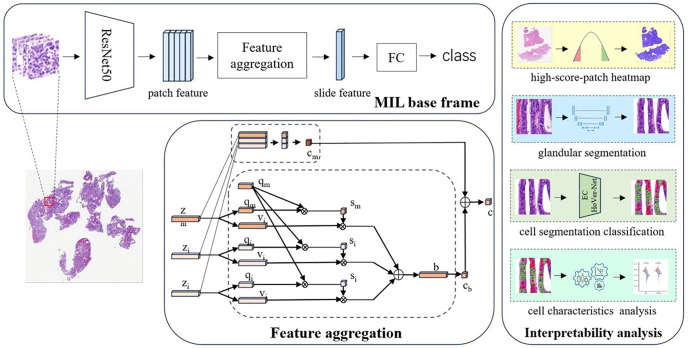
The main workflow of our research. The process is divided into three distinct phases. In the **WSI Pre-processing** phase, Whole Slide Images are preprocessed through background filtering and tissue extraction. Each WSI is then divided into a series of smaller patches for further analysis. In the **Multiple Instance Learning** phase, a multi-instance learning classification model is trained, and the attention scores from the model are used to identify the top 20 patches that are most relevant to the classification task. During the **Interpretability Analysis** phase, these top 20 patches are manually annotated for glandular regions, which are then used to train a segmentation network for automated gland segmentation. Tumor and non-tumor cells within the segmented images are subsequently manually labeled to train a nuclear segmentation network. Finally, morphological features such as cell nucleus count and size are extracted and analyzed to assess differences across various categories.

Our overall workflow framework is depicted in Fig 1. The approach is divided into three main stages. In the WSI pre-processing phase, the non-tissue regions of the slides, which do not contain useful information, are filtered out using the OTSU method. Subsequently, the remaining tissue regions are cropped into image patches of size 256 for feature extraction. In the MIL phase, the ResNet50 network is employed to extract texture and structural features of the cells within the patches, which play a crucial role in the model’s ability to distinguish between different categories. The features of the patch set are then aggregated using a feature aggregation model to generate a feature representation of the entire slide. Max pooling is applied to select the patches most likely associated with the target categories, and cosine similarity is computed between patches to establish inter-patch relationships. The aggregated slide-level features are used for category classification. In the interpretability phase, attention scores assigned based on cosine similarity are used to select the top 20 patches, which are then employed for glandular region segmentation and cell segmentation classification, thereby validating the accuracy of the model’s feature extraction.

#### 2.2.1. Multiple instance learning.

In multiple instance learning, the input slide X is a bag containing many instances, which corresponds to a pathological slide of the endometrium being cropped into a collection of patches. Features are extracted from small images through the convolutional neural network E(·), with the entire set of small images represented as $\text{X}=\{{\text{x}}_{\text{i}}{\}}_{\text{i}=\text{1}}^{\text{N}}$, xi represents the ith small image in the slide, and the features within the bag are represented as $\text{Z}=\{{\text{z}}_{\text{i}}{\}}_{\text{i}=\text{1}}^{\text{N}}$, where N is the number of patches in the slide, a variable value due to the uncertain size of tissues in the slide. In slide classification tasks, each slide has a known label Y ∈ C1, corresponding to the four categories in our task, EC、AEH、EH, and NE, and an unknown label for each instance yn ∈ C2. The goal of multiple instance learning is to use the instance features Z of the entire bag as input, and fit a function M(·) to predict the bag’s label Y. The common approach is to learn a feature aggregation function F(·), aggregate the features O of the bag from all instance feature sets Z within the bag, and then obtain the final predicted category Y’ through the classification function C(·), formally defined as follows:


M=C(F(E(X))),

#### 2.2.2. Feature extraction.

In the multiple instance learning framework, the feature extraction module for pathological slide classification is usually decoupled from the feature aggregation module. This decoupling is primarily due to memory constraints, as pathological slides typically have high resolutions, requiring substantial computational resources to extract features. Therefore, having a separate feature extraction module helps in flexibly controlling memory usage. Additionally, the initialization of the weights for the feature extraction module is an important consideration. Due to the lack of labels for the cropped patches, some studies have adopted pre-trained weights on large-scale datasets like ImageNet [26,27]. The ResNet network can extract tissue texture and morphological features of cells in pathology images. Building upon the success of these existing approaches, in this experiment, we utilized the pre-trained weights of the ResNet50 network on ImageNet to extract features from the patches.

#### 2.2.3. Feature aggregation.

The role of the special certification aggregation module is to aggregate the instance-level features obtained from feature extraction, thereby generating a slide-level feature representation that encapsulates inter-tissue relationships from cell-level features within each patch. The feature aggregation module used in this experiment was based on the design of the feature aggregation module in deep multi-instance learning model (DSMIL) [27]. It first utilizes maximum pooling to obtain the most relevant instance for classification. Then, it calculates the cosine similarity between this instance and other instances in the package. The similarity is used as the weight for the other instances. By summing the weighted sum of the instance feature values with their respective weights and the feature of the most relevant instance for classification, the feature of the entire package is obtained.

The formalization of extracting the most relevant instance for classification is as follows:


$${\text{c}}_{\text{m}}(\text{Z})=\text{max}\left({\text{W}}_{\text{0}}{\text{Z}}_{\text{0}},...,{\text{W}}_{\text{0}}{\text{Z}}_{\text{N}}\right),$$

Where ${\text{W}}_{\text{0}}$ It is a learnable weight vector, and it is used to identify the most relevant instance through maximum pooling. Then, we need to assign weights to the remaining instances in the slide to aggregate features and obtain the feature representation of the package. The weight assignment process is formalized as follows:


$$\text{U}\left({\text{z}}_{\text{i}},{\text{z}}_{\text{m}}\right)=\frac{\text{exp}\left(<{\text{z}}_{\text{i}},{\text{z}}_{\text{m}}>\right)}{\sum_{\text{k}=\text{0}}^{\text{N}}\text{e}\text{xp}\left(<{\text{z}}_{\text{i}},{\text{z}}_{\text{m}}>\right)},$$

The weights are assigned to the instances by calculating the cosine similarity between each remaining instance and the most relevant instance for classification. This formalizes the process of weight assignment as follows:


$${\text{c}}_{\text{b}}(\text{Z})={\text{W}}_{\text{b}}\sum_{\text{i}}^{\text{N}}\text{U}\left({\text{z}}_{\text{i}},{\text{z}}_{\text{m}}\right){\text{z}}_{\text{i}},$$

${\text{W}}_{\text{b}}$is a learnable classification weight. The final feature of the package is composed of two parts: the aggregation of the most relevant instance for classification, and the aggregation of the remaining instances:


$$\text{C}(\text{B})=\frac{\text{1}}{\text{2}}\left({\text{W}}_{\text{0}}{\text{z}}_{\text{m}}+{\text{W}}_{\text{b}}\sum_{\text{i}}^{\text{N}}\text{U}\left({\text{z}}_{\text{i}},{\text{z}}_{\text{m}}\right){\text{z}}_{\text{i}}\right)$$

#### 2.2.4. Interpretability verification.

To reveal the interpretability of the slide classification model, we conducted an in-depth analysis of the key regions the model focuses on. First, we extracted the top 20 patch sets from each slide using the patch attention scores in the feature aggregation network of the slide classification model as the main factor. Then, these patch sets were segmented into glandular regions using nnU-Net V2 [28]. Compared to nnU-Net V1, nnU-Net V2 introduces a deeper network that can capture more complex features, incorporates mixup data augmentation methods, and still dynamically adjusts the model’s structure and learning process. Next, pathologists annotated the normal endometrial glandular epithelial cells and atypical endometrial glandular epithelial cells within the glandular regions of the patches. The HoverNet model is trained and tested using the annotated images [29]. We apply the trained model to segment all top-20 patches in the entire set of WSIs.

To further reveal the differences in cellular-level features among different categories of endometrial tissue extracted by the model, we calculated cellularity and nuclear morphology based on pathologists’ clinical diagnostic experience and cell segmentation results, including cell proportion, cell density, circularity, area, perimeter, and axis ratio. Cellularity is defined as the percentage of cells’ area compared to the total area [30]. Each nucleus was measured on both long and short axes, and the long-to-short axis ratio was calculated. Cell density was measured by dividing the number of cell subtypes by the area of the cells [31]. The Mann-Whitney U test (Wilcoxon rank sum test) was used for comparison between groups. *P*-value < 0.05 was considered statistically significant.

## 3. Results

### 3.1. DSMIL performance evaluation in endometrial lesions classification

We evaluated the DSMIL model’s classification performance in the four-category classification task for endometrial lesions. For the experiments, we chose the Adam optimizer, with a batch size of 1 for the MIL classifier, an initial learning rate of 1 × 10^−4^, and a decay factor of 0.5 every 10 epochs. The slide block classifier had a batch size of 64, an initial learning rate of 5 × 10^−5^, and a decay factor of 0.5 every 10 epochs.

**Fig 2:**
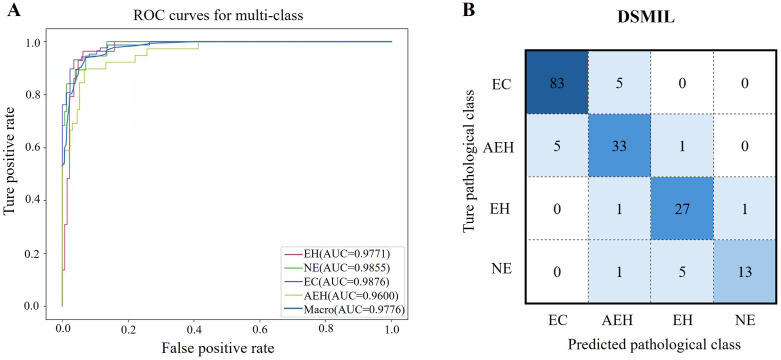
DSMIL model performance evaluation in endometrial lesions classification. (A) Model average ROC curve and inter-class ROC curves. (B) Confusion matrix associated with the multi-instance classification model. The darker the diagonal color, the higher the correct classification rate. Abbreviation: Endometrial carcinoma (EC), atypical endometrial hyperplasia (AEH), endometrial hyperplasia without atypia (EH), normal endometrium (NE).

Our multi-instance method achieved a macro-average AUROC of 0.9776 on the test dataset. For each pathological category, the AUROCs were 0.9876 for EC, 0.9600 for AEH, 0.9771 for EH, and 0.9855 for NE (Fig 2A). The confusion matrix indicates the true category of endometrial tissue and the predicted category based on images of the test dataset (Fig 2B). According to the test set results, the DSMIL model correctly classified the majority of endometrial pathological sections. Table 1 shows the macro-average performance and inter-class performance evaluation of this model. DSMIL had an average accuracy of 0.8914 on the test set. In the inter-class classification task, EC accuracy was 0.9429, AEH was 0.9257, EH was 0.9543, and NE was 0.9600. All of these results indicate that the DSMIL model is valid and reliable for diagnosing endometrial lesions.

**Table 1:** Performance metrics results of the DSMIL model in classifying endometrial lesions on test sets.

Class	AUROC	Accuracy	F1-score	Precision	Recall	Sensitivity	Specificity
Macro-average	0.9776	0.8914	0.8594	0.8787	0.8511	0.8511	0.9609
EC	0.9876	0.9429	0.9432	0.9432	0.9432	0.9432	0.9425
AEH	0.9600	0.9257	0.8354	0.8250	0.8462	0.8462	0.9485
EH	0.9771	0.9543	0.8710	0.8182	0.9310	0.9310	0.9589
NE	0.9855	0.9600	0.7879	0.9286	0.6842	0.6842	0.9936

### 3.2. Comparison of classification schemes

Our study compared the proposed DSMIL with other algorithms used to diagnose endometrial histological lesions. We conducted comparative experiments with other glandular lesion diagnosis models, including TransMIL, CLAM, and ABMIL, to examine similarities and differences between the DSMIL model and other neural network models. TransMIL re-encodes the extracted patch features using a transformer architecture model, CLAM predicts the patch categories and aggregates the slide labels accordingly, and ABMIL utilizes a fully connected network to learn the instance scores for feature aggregation.

**Fig 3:**
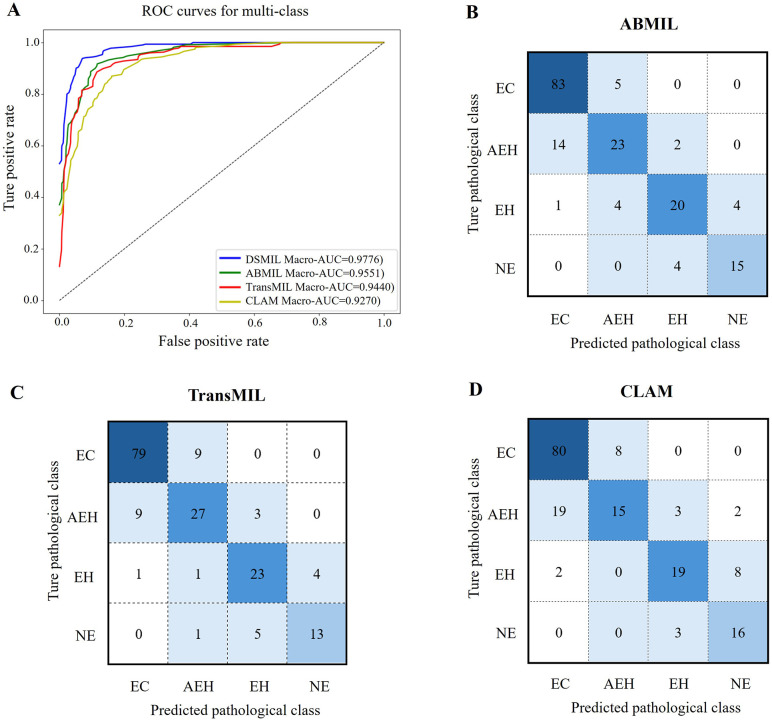
Comparison of average ROC curve (A) and confusion matrices for classification of endometrial lesions by different models on test sets, including DSMIL, (B) ABMIL, (C) TransMIL, and (D) CLAM models.

Table 2 presents the results of different methods of diagnosing endometrial lesions. DSMIL performed the best in the four-classification task with a diagnostic accuracy of 0.8914 and an AUROC of 0.9776, which was significantly higher than the other three methods (Fig 3A). DSMIL and ABMIL performed significantly better than the other two classifiers in AUROC. Fig 3B–D showed the confusion matrix for the experimental data set in the four-classification test. In comparison with TransMSL and CLAM, DSMIL had lower misclassification rates for identifying EC, AEH, and EH. In addition, DSMIL had lower misclassification rates in identifying AEHs and EHs than ABMIL.

**Table 2:** Performance comparison of different models in classifying endometrial lesions on test sets.

Algorithms	Accuracy	AUROC	F1-score	Precision	Recall	Sensitivity	Specificity
DSMIL	0.8914	0.9776	0.8594	0.8787	0.8511	0.8511	0.9609
TransMSL	0.8114	0.9421	0.7707	0.7762	0.7668	0.7668	0.9309
CLAM	0.7429	0.9236	0.6863	0.7049	0.6977	0.6977	0.8986
ABMIL	0.8057	0.9825	0.7643	0.7811	0.7530	0.7530	0.9237

### 3.3. Model interpretability in endometrial lesions classification

This study also examined visualization and interpretability, generating attention heatmaps for the WSIs. Using the attention score of each patch, we intuitively explained the importance of each area in the WSIs for the model to predict the classification of endometrial lesions. Areas with high attention scores corresponded to areas with typical pathological morphological features, while areas with low attention scores corresponded to areas with non-pathological features.

**Fig 4:**
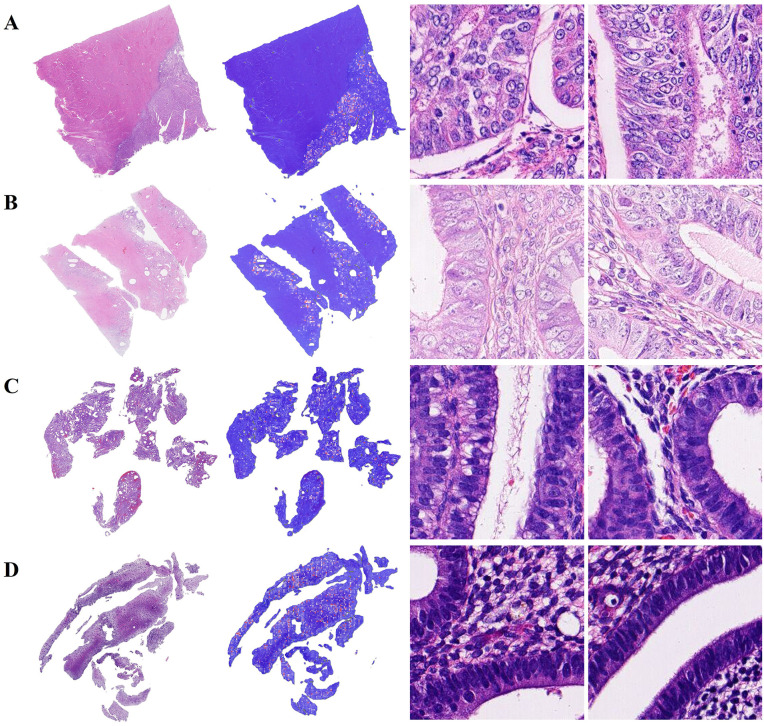
Interpretability and visualization of human endometrial tissue slices. Correctly classified WSIs heat maps of representative (A) EC, (B) AEH, (C) EH, and (D) NE tissue slices. From left to right are the original WSIs, the prediction heat maps, and the patches with high attention scores.

DSMIL macro-average accuracy reached 0.8914. Based on the results, the model accurately identified regions of diseased and non-diseased tissue on most endometrial WSIs and had excellent interpretability. H&E-stained WSIs of representative human endometrial tissue samples with correct slide-level classification are shown in Fig 4.

**Fig 5:**
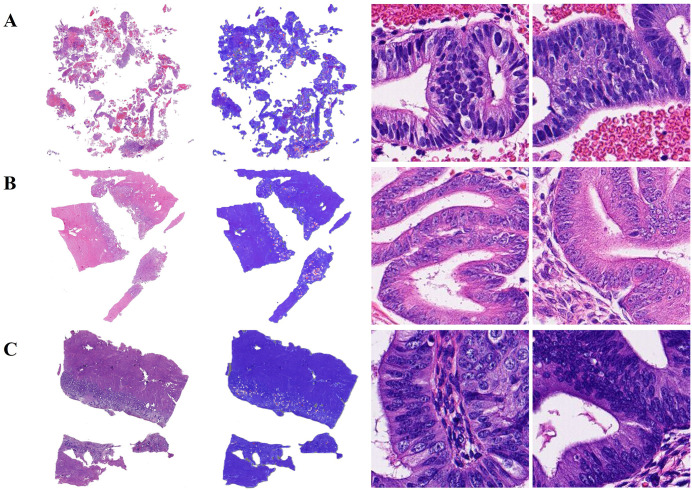
Incorrectly classified WSIs heat maps, for example, human endometrial tissue slices. (A) The actual NE WSI was classified as EH. (B) The actual AEH WSI was classified as EC. (C) The actual EC WSI was classified as AEH. From left to right are the original WSIs, the prediction heat maps, and the patches with high attention scores.

Additionally, the inter-class accuracy of EC, AEH, EH, and NE reached 0.9429, 0.9257, 0.9543, and 0.9600, respectively. Next, we analyzed the DSMIL model’s misclassification of endometrial tissue. There were five NE tissues misidentified as EH in the test set, possibly due to the crowded arrangement of glands in the segmented local patch images (Fig 5A). The confusion between AEH and EC tissue classifications may be due to the limited display and complex arrangement of glands within patch images, which makes it difficult for the model to accurately determine the integrity of the glandular basement membrane (Fig 5B, C).

### 3.4. Interpretability of local phenotypes

We extracted the top 20 patches with the highest attention scores in each WSI to explain pathological correlations based on local glands and cell regions. We utilized nnU-Net v2 to segment glandular tissue in the patch of endometrial tissue, employing its open-source method and default parameters for training. In the test set, the Dice reached 0.9438, indicating a high level of glandular segmentation. Subsequently, we used the HoverNet model to further segment atypical and normal glandular epithelial cells in the glandular area and also trained the model with default parameters. The Dice of test set segmentation reached 0.9304, and classification accuracy reached 0.8913, indicating effective cell segmentation. S1 Fig shows the segmentation areas of endometrial glands and endometrial glandular epithelial cells.

**Fig 6:**
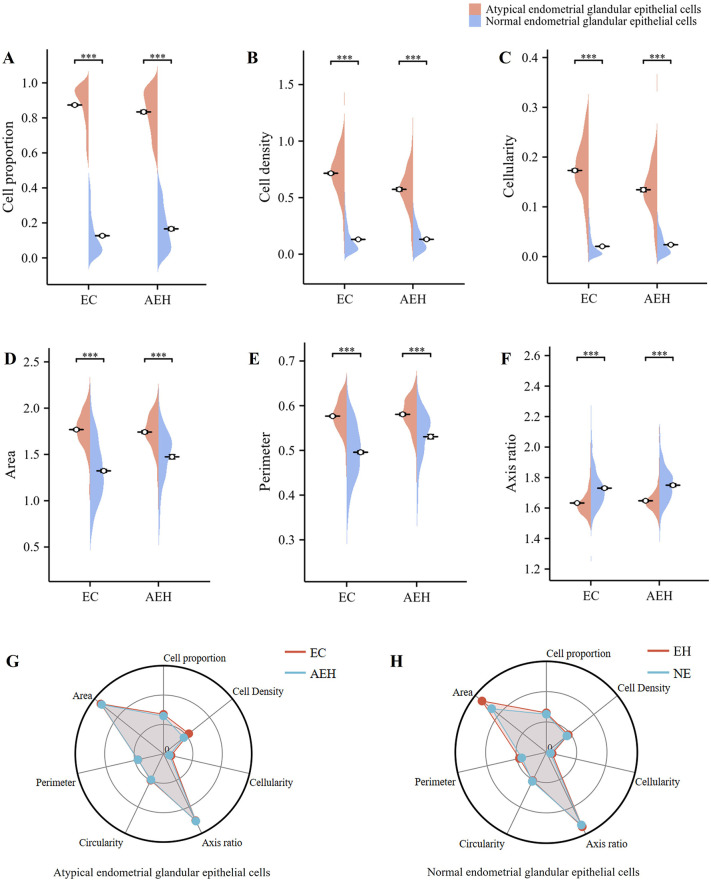
Structural and morphological characteristics of glandular epithelial cells in endometrial tissue. In EC and AEH tissues, atypical glandular epithelial cells had increased (A) cell proportions, (B) cell density, (C) cellularity, (D) area, (E) perimeter, and decreased (F) axis ratios than normal glandular epithelial cells. ***P < 0.001. (G) In EC and AEH tissues, atypical glandular epithelial cells were not significantly different in distribution and morphological characteristics. (H) In EH and NE tissues, normal glandular epithelial cells were not significantly different in distribution and morphological characteristics.

In both EC and AEH tissues, our model could accurately distinguish atypical glandular epithelial cells from normal glandular epithelial cells. Compared to normal glandular epithelial cells, atypical cells had increased cell proportions, density, cellularity, area, perimeter, and decreased axis ratios (Fig 6A–F). The morphological characteristics and distribution of atypical glandular epithelial cells in EC and AEH tissues were not significantly different (Fig 6G). Normal glandular epithelial cells of EH and NE did not differ significantly in morphological characteristics (Fig 6H). The results show that the nnU-Net v2 combined HoverNet model was significantly superior in distinguishing between atypical glandular epithelial cells and normal glandular epithelial cells.

## 4. Discussion

In summary, we propose an interpretable weakly supervised deep learning pipeline for the diagnosis of four-class endometrial lesions from digitized HE-stained WSIs. Our deep learning model was constructed, validated, and tested on 885 slides of 461 examples of endometrial tissue. Compared with other glandular classification models, our proposed DSMIL model achieved quite high performance in the four-category classification task for endometrial lesions diagnosis. In addition, we generated interpretative heat maps and extracted the top 20 patch images of each section with the highest representative pathological features. We also analyzed the correlation between pathological classification and endometrial glandular epithelial cell features. The nnU-Net v2, combined with the HoverNet model, has significant advantages in distinguishing atypical glandular epithelial cells from normal glandular epithelial cells. This study indicates that the DSMIL model is an efficient and accurate tool for auxiliary diagnosis of endometrial lesions, facilitating the accurate clinical shunt of patients requiring surgical treatment, and reducing the rate of misdiagnosis and missed diagnoses.

We present a weakly supervised learning workflow that demonstrated excellent accuracy in the four-class classification task of diagnosing endometrial lesions. An attention-based image classification model is combined with a weakly supervised multiple instance learning approach to learn the specific morphological features associated with each pathological category, which ultimately serves to diagnose endometrial pathology. Our DSMIL algorithm takes advantage of relationships between instances to assign attention weights, which are then used to aggregate slide features [27]. Compared to other gland classification models, such as TransMIL, CLAM, and ABMIL, DSMIL’s advantage is that the aggregated slide features are more comprehensive. This allows the integration of more instance features for classification prediction. According to the results, the proposed DSMIL offers significant potential for automatic endometrial lesion diagnosis.

We used visual heat maps to identify areas of endometrial tissue sections with the most significant pathological features. This was to determine the interpretability of the model for endometrial lesion diagnosis. The top 20 patches with the highest attention scores under this algorithm were further analyzed to determine the pathological correlation of diagnosis. For correctly classified tissue slices, the model captured features in local patches consistent with human visual assessments. For misclassified sections, patch images also capture the most critical pathological areas. However, since tissue patch images only show some prominent areas in WSIs, the analysis of the global characteristics of the glands is limited. This may be a major reason for classification confusion.

A combination of nnU-Net v2 and HoverNet models was used to identify glandular epithelial cells in the endometrial glandular region to further investigate the taxonomic correlation of local pathological tissue regions. Results showed that these models effectively identified atypical glandular epithelial cells from normal ones. Atypical glandular epithelial cells in EC and AEH tissues also showed significant consistency in our analysis. For endometrial tissues, EIN histological classification is commonly used in clinical analysis of nuclear and structural features [32]. According to the EIN system, the D-score was developed by integrating matrix volume percentage, glandular outer surface density, and standard deviation of the shortest glandular nucleus axis [33,34]. Incorporating the D-score into our deep learning system, which may predict the risk of AEH progressing to EC, will further enhance diagnostic efficiency and interpretability.

This study still has some limitations. First and foremost, because of the characteristics of hysteroscopic sampling in some cases, the tissue morphology of normal specimens is more likely to be unevenly distributed and broken than that of tumor tissues, increasing the difficulty of diagnosing such conditions. Furthermore, it is essential to conduct further studies to predict the risk of AEH developing into EC based on regional morphological characteristics. As part of our long-term plan, we intend to conduct multicenter clinical studies to expand tissue sample size and further optimize the endometrial disease diagnosis model.

Several prior studies have applied deep-learning approaches to predict molecular characteristics from H&E-stained whole-slide images and provide important context for our work. For example, the multi-resolution Panoptes framework has achieved multi-task prediction of histological subtypes, molecular classes, and common gene mutations in EC [17]. More recently, im4MEC integrated self-supervised learning with an attention mechanism to enable accurate classification of the four molecular subtypes of EC [24]. In addition, architecture based on an attention module has been successfully applied to MSI prediction in multiple tumor types, further underscoring the promise of this direction [35]. These studies mainly focus on the molecular classification of endometrial cancer. Compared with these studies, our work not only attains high identification performance in EC but also incorporates precancerous and normal endometrial tissues. By focusing on the continuum from normal endometrium to benign lesions and malignant transformation, our model aims to support earlier identification of pathological changes that may reduce the risk of progression to EC. Furthermore, the lightweight attention architecture used in our approach substantially reduces computational cost while preserving predictive accuracy. The simple and transparent slide-level aggregation strategy offers a more practical alternative to complex multi-stage inference pipelines. Combined with intuitive attention heatmaps, our method enhances interpretability and aligns more closely with real-world clinical pathology workflows. Overall, this study complements existing research by achieving a practical balance among model performance, interpretability, and feasibility for clinical deployment.

Beyond model performance, several practical considerations need to be carefully evaluated before DSMIL can be implemented in routine clinical settings. First, although DSMIL requires substantial computational resources during training, its inference process is relatively lightweight. To enhance deployment feasibility, future work will investigate model compression, lightweight feature extraction, and optimized inference pipelines to support efficient on-premise deployment within hospital infrastructures. Variability in staining protocols, scanner hardware, and diagnostic conventions across centers may also affect model robustness. To strengthen generalizability, we plan to conduct prospective multi-center evaluations to assess the model’s stability and applicability across diverse populations and routine clinical workflows. Second, model interpretability remains essential for pathologists without technical backgrounds. Attention-based heatmaps, instance-level scoring, and prototype visualization demonstrate good correspondence with conventional morphological features, helping clinicians understand the rationale behind model predictions and improving trust and acceptance. We will further explore more intuitive visualization interfaces and standardized reporting formats to facilitate seamless integration into digital pathology systems. Third, privacy considerations are particularly important for whole-slide image data. Clinical deployment requires compatibility with existing digital pathology infrastructure, and federated learning frameworks provide a secure paradigm for multi-institutional model development without sharing raw patient data [36]. These characteristics collectively enhance the feasibility of deploying DSMIL-based systems in clinical settings. In addition, we aim to investigate practical strategies for workflow integration, with emphasis on operational feasibility, usability, and acceptance in real clinical environments.

## 5. Conclusions

Our study proposes an interpretable weakly supervised deep learning pipeline that uses HE-stained WSIs to diagnose endometrial lesions, including EC, AEH, EH, and NE. This digital pathology workflow has great clinical application potential, as it serves as an efficient, rapid, and accurate auxiliary diagnostic tool for endometrial diseases, facilitates the accurate clinical shunting of patients requiring surgical treatment, and reduces the likelihood of misdiagnosis and missed diagnoses. We will also conduct large-scale, multi-center studies to validate this adjuvant approach, which is essential for clinical application.

### Consent for publication

All the authors agreed to be published.

## Supporting information

S1 Fig Representative images of endometrial glandular and glandular epithelial cell segmentation in human endometrial tissue WSIs, including (A) EC, (B) AEH, (C) EH, and (D) NE. From left to right are the patches with high attention scores, glandular segmentation images, and glandular epithelial cell segmentation images. The red circle shows atypical glandular epithelial cells identified by the algorithm, and the green circle shows normal glandular epithelial cells identified by the algorithm.
(PDF)

## Abbreviations

EC: Endometrial cancer
WHO: World Health Organization
EH: endometrial hyperplasia without atypia
AEH: atypical endometrial hyperplasia
AI: Artificial intelligence
MIL: multiple instance learning
NE: normal endometrial
WSIs: Whole-slide images
HE: hematoxylin and eosin.
