# Supplementary figures and images for: Weakly supervised deep multi-instance learning for classification of endometrial lesions on hematoxylin and eosin-stained whole-slide images
Source: PLoS One. 2026 Jan 2;21(1):e0340186. doi: 10.1371/journal.pone.0340186 (PMC12758718; doi:10.1371/journal.pone.0340186)

**A**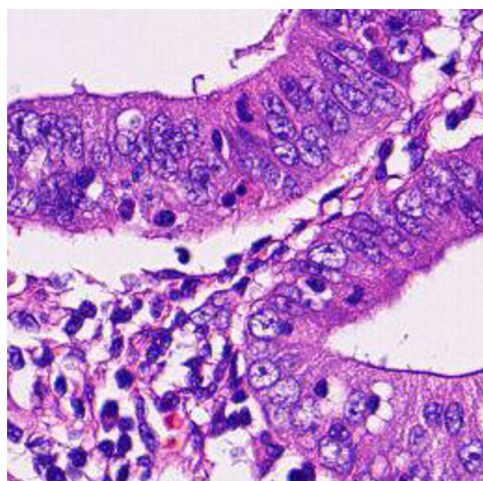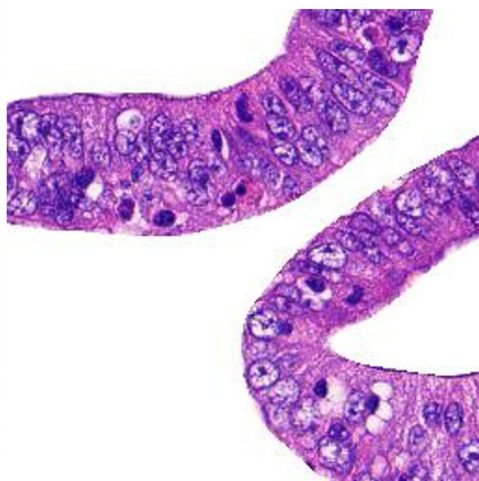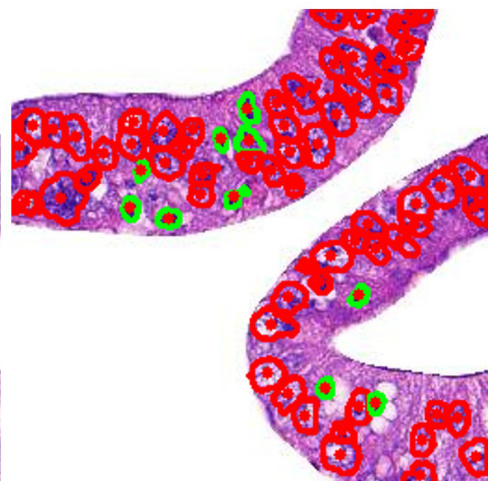**B**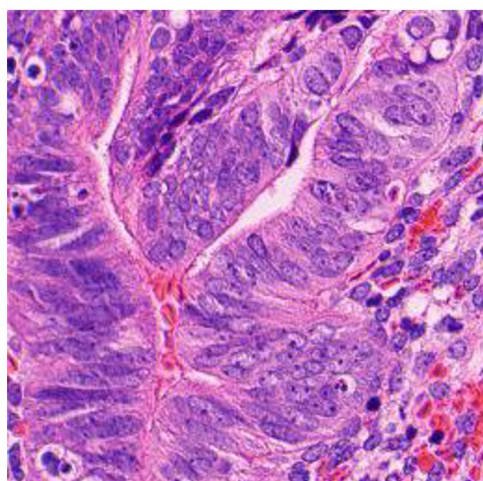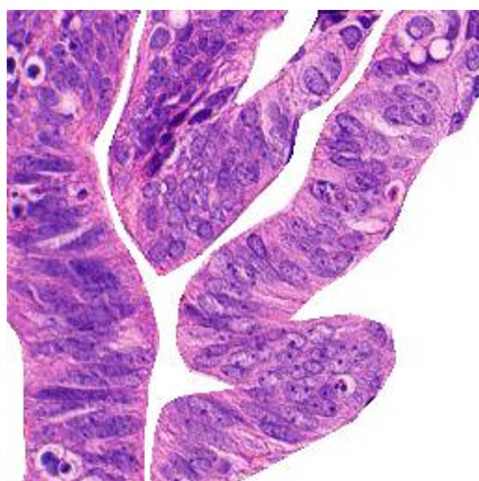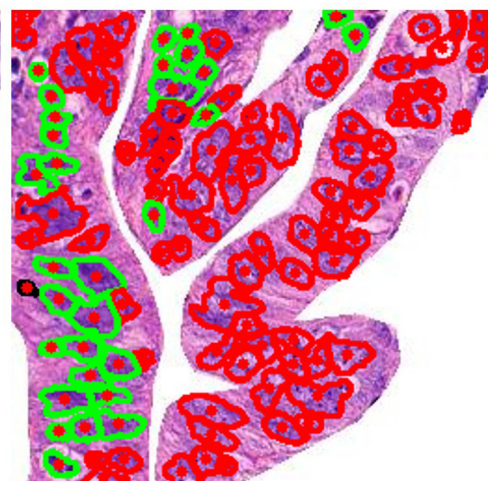**C**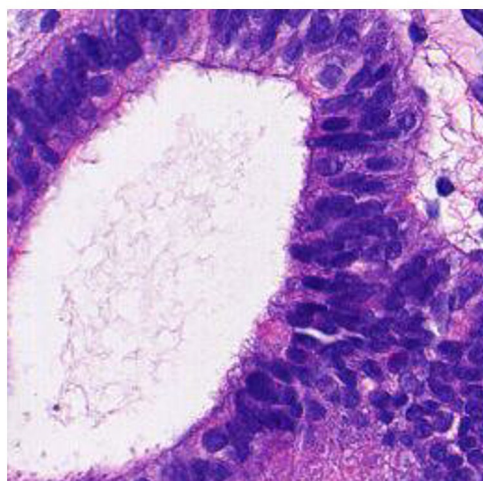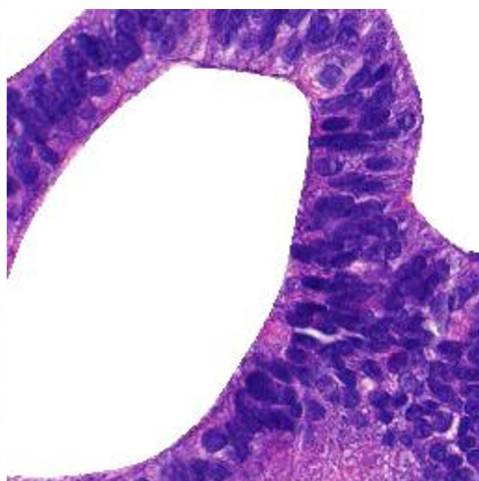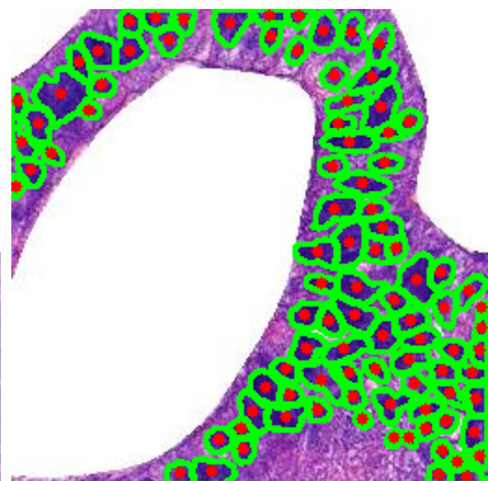**D**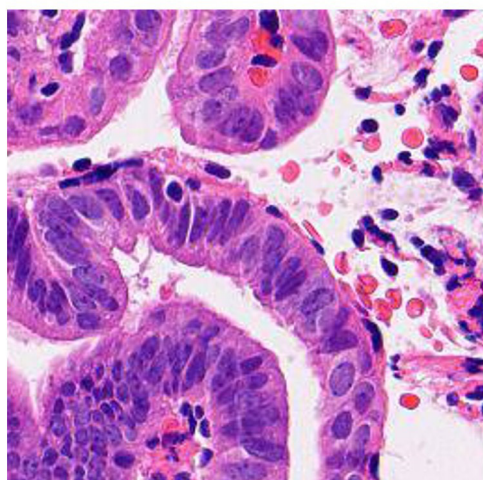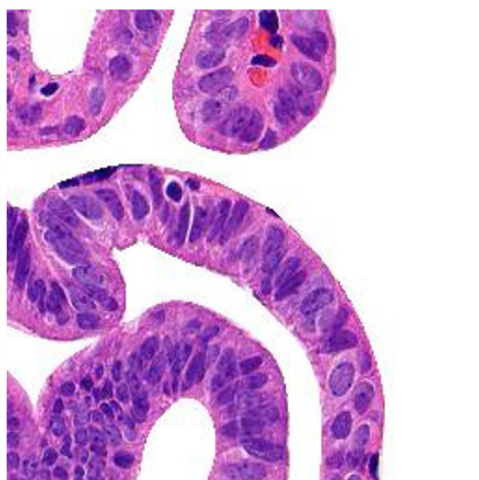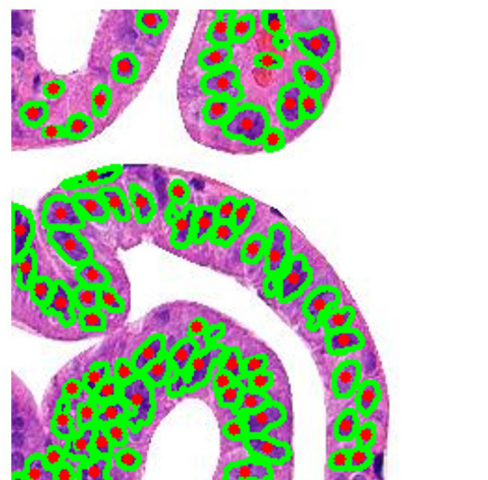

Supplement: S1 Fig — From left to right are the patches with high attention scores, glandular segmentation images, and glandular epithelial cell segmentation images. The red circle shows atypical glandular epithelial cells identified by the algorithm, and the green circle shows normal glandular epithelial cells identified by the algorithm. (PDF) [file pone.0340186.s001.pdf]
